# Ontario Veterinary College

**Published:** 1895-04

**Authors:** 


### COMMENCEMENT EXERCISES.

### ONTARIO VETERINARY COLLEGE.

THE twenty-sixth annual commencement of the Ontario Veterinary College was held in Assembly Hall, Toronto, on March 29. The number of graduates reached 150. The exercises were presided over by Prof. Andrew Smith, principal of the college. Among those present were the Lieutenant-Governor, Rev. Principal Caven, Dr. Cowan, of Galt; Mr. Robert Jaffray, Dr. Wilson, of London; Mr. Sisson, of the Ontario Agricultural and Arts Association; Dr. Daniel Clark, Mr. William Christie, Dr. Duncan, Commander Law, Mr. J. J. Winthrow, Mr. Henry Wade, Rev. Charles Campbell, Dr. May, Mr. H. J. Hill, Dr. Thorburn, Dr. Elliot, of St. Catharine's; Mr. C. H. Sweetapple, Mr. Septimus Sisson, C. G. Richardson, and Col. D'Arcy Boulton, of Cobourg.

Dr. Smith, referring to the college history from 1866, said there had been a gradual increase in the number of students until a period two years ago, since when there had been a falling off, though a greater extent of country was represented.

Dr. Duncan read the following list of graduates, honor and prize men:

Leslie J. Allen, Blue Mound, Kansas; James H. Armstrong, London, Ont.; C. E. Arnold, Brant, Ohio; Jas. E. Alyed, Consecon, Ont.; Oscar J. Amey, Ernestown Station, Ont.

Forrest P. Barrett, Peterborough, N. H.; James A. Bean, Wellington, Ont.; Llewellyn W. R. Best, Sandford, Ont.; S. P. Bishop, Greencastle, Pa.; Albert M. Blackwell, Omaha, Neb.; J. F. Blinzley, Norwalk, Ohio; David R. Bone, Paisley, Ont.; Edward T. Bowers, Cedar Rapids, Neb.; Henry Boyd, London, Ont.; O. E. Brandreth, Strathroy, Ont.; Charles E. Broad, Nomence, Ill.; Thomas Broom, Blenheim, Ont.; George J. Brossard, Fall River, Wis.; D. Clifton Burnett, Youngstown, Ohio; Edward D. Burns, Farmington, N. Y.; James H. Burt, Lostwithiel, Cornwall, England; Herman Busman, Cooperville, Mich.; Edward F. Bettinger, Chittenango, N. Y.; Ernest C. Burdick, Livonia, N. Y.

Harvey H. Carter, Brookfield, Ohio; John M. Coates, Kearney, Mo.; Frederick W. Cook, Jamesville, N. Y.; Dillon Coristine, Watford, Ont.; Aaron Cornell, Elkton, Mich.; Frank B. Cotton, Grand Rapids, Mich.; H. S. Creighton, Americus, Kansas; Bradford D. Curtis, North Attleborough, Mass.; Dwight S. Church, Scranton, Pa.

Willard De Bolt, Union City, Ind.; Roy E. Davis, Toledo, Ohio; James Dent, Milton, Ont.; J. M. Dodge, Caro, Mich.; Joseph A. Douglas, Blake, Ont.; William F. Duffield, Pittsfield, Ill.; W. J. Davis, Decatur, Ill.

Louis H. Eckert, Sebringville, Ont.

John W. Farr, Jarvis, Ont.; W. H. Farrow, Auburn, Ont.; George H. Fitch, Claremont, N. H.; George D. Fortune, Wingham, Ont.; William Fotheringham, Grenfell, N. W. T.; Arthur P. Freeman, Barrie, Ont.

James G. Galloway, Uxbridge, Ont.; Augustine J. Gibbons, Wingham,

Ont.; John G. Gibson, Alameda, N. W. T.; S. Wells Gibson, Cleveland, Ohio; H. M. Gillian, Ackley, Iowa; Francis W. Gokey, Guelph, Ont.; Albert E. Goodwin, Hager City, Wis.; Robt. L. Graham, Schomberg, Ont.; James Grant, Teeswater, Ont.; Lawrence A. Grinnell, Grand Ledge, Mich.

Joseph A. Hackett, Hockley, Ont.; Henry H. Hackler, Pottstown, Pa.; Joseph Hamilton, Auburn, Ont.; William S. Hamilton, Chelsea, Mich.; Edward T. Handy, Onondaga, Mich.; William H. Haney, Forest, Ont.; Bernard Harmon, Decorah, Iowa; George A. Hay, Campbellford, Ont.; Edwin M. Henry, Oswego, Kansas; Alfred S. Henhoffer, Strasburg, Ont.; Robert J. Henry, Mono Mills, Ont.; Robert C. Hill, Twin, Ohio; W. Proctor Hill, Frederick, Maryland; John B. Hollenbeck, Rock Valley, Iowa; Maurice A. Hollingsworth, La Salle, Ill.; David Hood, Alliston, Ont.; Roy White Hooker, Lowell, Mich.; Benj. F. Hoover, Davis, Ill.; William R. Hynes, New York City; Hiram E. Hensel, Arcadia, Wis.

Charles E. Inskeep, Port Jefferson, Ohio; George Ireland, Inglewood, Ont.

Albert W. James, Toronto, Ont.; John V. Jewell, Marcus, Iowa; John W. Johnson, Bath, Ont.

J. M. Klinck, Toronto.

William H. Lake, Miami, Man.; Alexander O. Lee, Toronto; John Letham, Stonehouse, Scotland; Don. J. Loofbourrow, Mt. Sterling, Ohio; Robert C. Lipsett, Glenboro, Man.; George A. Lipsett, Glenboro, Man.

Fred. S. McA. Macdonald, Dundas, P. E. I.; Robert Webber McKibbin, Buck Valley, Pa.; Frank J. McNeal, Shickshinny, Pa.; Harry T. McNeal, Shickshinny, Pa.; W. Albert McNeely, Green River, Ont.; Colin McPherson, Glanworth, Ont.; John McKay, Duluth, Minn.; Donald McKercher, Petrolea, Ont.

Fred. De Witt Markham, Port Leyden, N. Y.; Claude L. Megowan, Sacramento, Cal.; Peter J. Mertz, Virginville, Pa.; Richard A. Milne, Don, Ont.; J. J. Moore, Corning, Iowa; George F. Myers, Danville, Ill.; Henry H. Myers, Youngstown, Ohio; Alfred A. Moody, Guelph, Ont.; William M. Morris, Cass City, Mich.

William Nicholson, Pittsburg, Pa.; Robert R. Nickell, Limehouse, Ont.

W. H. Oatway, Chicago, Ill.

Daniel George Peat, Plainville, Ont.; Albert M. Perdue, Wingham, Ont; Richard M. Phelan, Greenville, Pa.; Walter R. Pick, Columbus, Wis.; Wm. T. Pugh, Grafton, N. Dakota.

Edward Rafter, Hamburg, N. Y.; G. G. Ratz, Red Bud, Ill.; Charles M. Roche, Cork, Ireland; Peter J. Roets, Monches, Wis.; Henry C. Riddle, Waterloo, Ont.

J. R. Saxton, Strathroy, Ont.; M. J. Sexton, Minneapolis, Minn.; Arthur L. Shaw, Mt. Upton, N. Y.; Franklin Gifford Shepard, North Evans, N. Y.; William Morris Stanley, Guelph, Ont.; Henry D. Stebbins, Westmoreland, N. Y.; W. Oscar H. Stener, New Haven, Conn.; Archibald D. Stewart, Ailsa Craig, Ont.; James Stewart, Ailsa Craig, Ont.; Thomas M. Sweeny, Richmond, Va.; M. B. Stiver, Mount Albert, Ont.

James A. Tancock, London, Ont.; James W. Thompson, Cameron, Mo.; J. Thomson, Clinton, Iowa; Geo. P. Tucker, Lincoln, Neb.; Horace Bruce Taylor, Nicholasville, Ky.; Cæsar Augustus Turley, Brandon, Man.

Ernest George Vans Agnew, Fulda, Minn.; Frank Vans Agnew, Rochester, Minn.; Robert Vans Agnew, Fulda, Minn.; Edward F. Vorhis, Ithaca, New York.

Thomas G. Waghorn, Kirkton, Ont.; Duran M. Walco, Grand Ledge, Mich.; Edward K. Ward, Guilford, Ind.; John Welch, Strathroy, Ont.; William W. Welch, Elgin, Ill.; William Lincoln West, Ellsworth, Maine; Allan S. White, Glenallan, Ont.; Joseph Wingerter, Akron, Ohio; Hervey W. Witmer, Greencastle, Pa.; Clarence D. Wooding, Waterbury, Conn.; Alexander H. Wright, Columbus, Wis.; Frank W. Waterman, Providence, R. I.; Macy Weesner, Fort Wayne, Ind.; Fred. A. Wilson, Toronto, Ont.

Amos W. Young, North Liberty, O.; David F. Young, Alameda, N. W. T.

*Seniors by Departments.*

Pathology (seniors)—W. L. West, silver medal; W. Fotheringham, second prize; F. Vans Agnew, third prize. Honors—L. J. Allan, C. E. Arnold, F. V. Barratt, S. P. Bishop, E. F. Bettinger, E. T. Bowers, H. Boyd, O. E. Brandreth, G. J. Brossard, D. C. Burnett, E. D. Burns, J. H. Burt, E. C. Burdick, H. Busman, J. M. Coates, D. Coristine, H. S. Creighton, B. D. Curtis, W. De Bolt, W. J. Davis, Roy E. Davis, G. D. Fortune, W. Fotheringham, G. Galloway, A. C. Goodwin, R. L. Graham, L. A. Grinnell, H. D. Hackler, W. S. Hamilton, E. T. Handy, E. M. Hendy, A. S. Henhoeffer, R. C. Hill, H. E. Hensell, J. B. Hollenbeck, M. A. Hollingsworth, A. O. Lee, J. Letham, D. J. Loofbourrow, R. W. McKibben, H. T. McNeal, D. McKercher, F. De W. Markham, C. L. Megowan, G. Myers, R. R. Nickell, W. H. Oatway, R. M. Phelan, E. Rafter, H. C. Riddle, F. G. Shephard, H. D. Stebbins, A. D. Stewart, T. M. Sweeny, J. A. Tancock, H. B. Taylor, J. Thomson, G. P. Tucker, F. Vans Agnew, R. Vans Agnew, E. K. Ward, W. W. Welch, W. L. West, H. W. Witmer, C. D. Wooding, A. H. Wright, A. W. Young.

Materia Medica (seniors)—First prize, T. M. Sweeny; second prize, William Fotheringham; third prize, W. R. McKibbon. Honors—J. H. Burt, J. M. Farquhar, A. S. Henhoeffer, F. D. Markham, H. C. Riddle, F. Vans Agnew, W. L. West.

Chemistry (seniors)—First prize, William Fotheringham; second prize, T. M. Sweeny; third prize, E. Rafter. Honors—H. E. Hensel, W. R. Hynes, A. A. Moody, W. H. Oatway, G. G. Ratz, W. L. West.

Morbid Anatomy (seniors)—First prize, G. D. Fortune; second prize, W. L. West; third prize, W. Fotheringham. Honors—S. P. Bishop, Herman Busman, F. D. Markham, T. M. Sweeny, F. Vans Agnew.

Anatomy (seniors)—Silver medal, W. L. West; second prize, G. D. Fortune; third prize, W. Fotheringham and T. M. Sweeny, equal. Honors—L. J. Allen, J. H. Armstrong, C. E. Arnold, J. Brown, J. A. Bean, G. J. Brossard, H. Busman, D. C. Burnett, L. A. Best, O. E. Brandreth, E. F. Bettinger, J. Burt, J. M. Coates, B. D. Curtis, H. S. Creighton, D. Coristine, A. Cornell, J. A. Douglas, W. De Bolt, T. B. Cotton, W. F. Duffield, J. M. Farquhar, A. E. Goodwin, L. A. Grinnell, F. W. Gokey, J. G. Galloway, J. M. Gibson, R. L. Graham, E. M. Hendy, E. T. Handy, W. P. Hill, D. Hood, W. R. Hynes, W. Haney, W. S. Hamilton, A. Henhoeffer, R. C. Hill, J. Latham, P. J. Meetz, H. H. Myers, G. F. Myers, R. A. Milne, F. D. Markham, D. McKercher, W. McKibben, F. S. Macdonald, W. H. Oatway, W. Pugh, W. R. Pick, D. G. Peat, A. M. Perdue, G. G. Ratz, H. C. Riddle, N. M. Stanley, H. D. Stebbins, F. Sheppard, A. F. Shaw, H. B. Taylor, J. A. Tancock, J. W. Thompson, John Thomson, F. Vans Agnew, R. Vans Agnew, W. W. Welch, John Welch, H. W. Witmer, A. H. Wright.

Dissected Specimens (seniors)—A. A. Moody, gold medal, given by Toronto Industrial Exhibition Association; F. Vans Agnew, second prize, $30; E. Rafter, third prize, $20.

Entozoa (seniors)—A. E. Goodwin, first prize. Honors—L. J. Allan, S. T. Bishop, I. Burt, J. A. Douglas, W. Fotheringham, J. G. Galloway, R. T. Graham, E. J. Handy, H. E. Hensel, W. R. Hyams, A. Henhoeffer, R. Milne, P. J. Mertz, A. A. Moody, F. S. Markham, T. M. Sweeny, John Thomson, R. Vans Agnew, W. L. West.

Physiology (seniors)—First prize, silver medal, Frank Vans Agnew; second prize, T. M. Sweeny; third prize, William Fotheringham. Honors—L. J. Allen, S. P. Bishop, J. H. Burt, J. A. Douglas, G. D. Fortune, A. E. Goodwin, A. S. Henhoeffer, W. R. Hynes, H. C. Riddle, H. B. Taylor, J. Thomson, E. G. Vans Agnew, W. L. West.

Best general examination—T. M. Sweeny, gold medal. Honors—S. P. Bishop, H. Busman, F. D. Markham, H. D. Stebbins, F. Vans Agnew, W. L. West.

*Juniors by Departments.*

Pathology (juniors)—First prize, H. R. Ryder; second prize, E. W. Culley; third prize, H. Taylor, R. J. Stanclift, equal. Honors—W. Agnew, H. Agnew, F. Armstrong, J. F. J. Black, A. Brechter, A. C. Campbell, P. Cain, J. L., Clark, A. E. Dennis, R. A. Dennis, J. R. Donahue, C. H. Drake, A. F. Elliott, T. H. Ferguson, J. B. Finch, W. J. Grady, C. Haas, W. W. Hall, W. E. Howe, W. N. J. Hurt, L. Heyes, C. Inglis, R. E. Jackson, P. Johnson, J. E. Johnston, N. Kippen, R. Kjerner, J. Lawrason, W. S. Longacre, J. D. Macdonald, L. Mulligan, R. W. Macdonald, A. J. McKay, H. E. Marshall, G. R. C. Merriam, W. D. Monk, P. Palmer, J. D. Paxton, O. Reed, L. T. Richards, J. L. Riddell, P. Robinson, L. D. Ryan, T. W. Simpson, F. C. Simpson, J. S. Sparrow, C. E. Steele, D. A. Stewart, J. A. Stevenson, G. A. Wehr, M. A. Whimster, A. E. Williamson, D. B. Wilson, F. L. Wingate, C. Woods, A. R. Walsh, R. E. Willis, A. G. Young.

Anatomy (juniors)—Henry Taylor, silver medal; E. W. Culley, second prize; George Ingles and G. R. C. Merriam, equal for third prize. Honors—W. Agnew, C. H. Drake, A. F. Elliott, T. H. Ferguson, W. E. Howe, J. E. Johnston, W. Longacre, J. D. Paxton, F. S. Schneiderf, C. Steel, D. A. Stewart, Carlos Woods, J. F. J. Black, J. R. Donahue, J. B. Finch, L. Hay, W. W. Hall, R. E. Jackson, Peter Johnson, R. Kjerner, S. Mulligan, A. J. McKay, L. Richards, H. R. Ryder, J. Simpson, R. J. Stanclift, M. A. Whimster, A. C. Young.

Physiology (juniors)—First prize, silver medal, Geo. Ingles; second prize, G. R. C. Merriam; third prize, J. R. Donahue. Honors—J. F. J. Black, E. W. Culley, W. T. Howe, Newton Kippen, H. R. Ryder, T. V. Simpson, H. Taylor.

Chemistry (juniors)—First prize, Henry Taylor; second prize, J. F. J. Black; third prize, Leopold Hay and R. J. Stanclift, equal. Honors—John P. Fitsgerald, A. E. Johnson, T. Lockwood Wingate, W. D. Monk, L. T. Richards, L. D. Ryan, F. C. Simpson, Thos. V. Simpson, E. A. Williamson.

Histology and Microscopy—Juniors—First prize, R. J. Stanclift; second prize, J. F. J. Black; third prize, G. R. C. Merriam. Honors—William Agnew, John L. Clark, E. W. Culley, R. A. Dennis, J. R. Donahue, A. F. Elliott, T. H. Ferguson, John B. Finch, John P. Fitzgerald, W. J. Grady,

Charles Haas, George Inglis, Roy E. Jackson, Peter Johnson, J. E. Johnston, N. Kippen, Randolph Kjerner, John Lawrason, W. S. Longacre, R. W. McDonald, John D. Macdonald, H. E. Marshall, Walter D. Monk, Lemuel Mulligan, John D. Paxton, Llewellyn N. T. Richards, Louis D. Ryan, Francis C. Simpson, Thomas V. Smith, John J. Sparrow, C. E. Steele, Henry Taylor, M. A. Whimster, Arthur E. Williamson, F. L. Wingate, Carlos Woods, Alex. G. Young.

Primary Examinations—Anatomy—A. S. Berryman, F. J. De Land, F. Duncan, J. M. Farquhar, E. T. Gore, G. H. Metcalfe, A. A. McArthur, Angus McDonald, J H. Powers, A. M. Twohey, J. Wagner, E. C. Wisman.

Materia Medica—J. M. Farquhar, F. A. Fawcett, Allan McDonald, J. Nelson, J. H. Powers, C. E. Wilson.

The prizes and medals were awarded the successful students by the Lieutenant Governor, after which the graduates were addressed by Principal Cavin, Dr. Clark, Mr. Winthrow, and Dr. May.

Principal Cavin in his address spoke very cordially of the international relations that existed between the United States and Canada.

Dr. Clark, in his remarks referring to kindness to the lower animals, thought that the man who was not kind had some twist in his moral nature, and "if animals could talk as men talk even Balaam's ass would pale his ineffectual fires."

Mr. Stebbins, of Westmoreland, N. Y., on behalf of the students, presented Dr. Smith with a fine group picture of the graduating class of 1895.

---

